# Rapid Intervention to Support Eating Issues (RISE) Program: Using Quality Improvement to Reduce Medical Hospitalization in Malnourished Youth

**DOI:** 10.1111/eat.70004

**Published:** 2025-11-18

**Authors:** Michele Calderoni, Samantha Turner, Kerri Heckert, Carrie Snyder, Nicole Cifra, Rebecka Peebles, Karen Foy, Jonathan Walsh, Amanda O. Widders, C. Alix Timko

**Affiliations:** ^1^ Craig‐Dalsimer Division of Adolescent Medicine, Children's Hospital of Philadelphia Philadelphia Pennsylvania USA; ^2^ University of Pennsylvania Perelman School of Medicine Philadelphia Philadelphia Pennsylvania USA; ^3^ Department of Nursing University of Massachusetts Boston Boston Massachusetts USA; ^4^ Department of Clinical Nutrition Children's Hospital of Philadelphia Philadelphia Pennsylvania USA; ^5^ Main Line Center for Eating Disorders Philadelphia Pennsylvania USA; ^6^ Monte Nido Miami Florida USA; ^7^ Lehigh Valley Health Network Allentown Pennsylvania USA; ^8^ Department of Child and Adolescent Psychiatry and Behavioral Sciences Children's Hospital of Philadelphia Philadelphia Pennsylvania USA

**Keywords:** early intervention, eating disorder, focused, malnutrition, program‐led, quality improvement

## Abstract

**Objective:**

Program‐led and focused models may overcome structural barriers to accessing ED care, such as limited availability, for youth with EDs by prioritizing strategic, evidence‐based care delivered through a structured approach. The Rapid Intervention to Support Eating Issues (RISE) pilot aimed to promote weight restoration and prevent hospitalization among malnourished adolescents at risk for hospitalization. We used a “home hospital” approach, integrating medical oversight, family‐based treatment principles, and nutritional support via structured outpatient care.

**Methods:**

Participants completed 4–5 visits with adolescent medicine and nutrition over 8 weeks. They received psychoeducation and support in implementing home hospital. Vital signs, anthropometrics, dietary intake, ED behaviors, and cognitions were assessed.

**Results:**

A total of 27 patients participated. Patients experienced low hospitalization rates (*n* = 1 throughout; 3.7%) and significant weight gain (M_end of treatment_ = +2.7 kg from baseline, 95% CI: 2.6–4.7). There were statistically significant increases in calorie intake (M_baseline_ = 43.3% of recommendation; M_end of treatment_ *=* 76.0% of recommendation; *dz* = 0.98, 95% CI: 0.45–1.50) and decreases in the report of disordered weight control behaviors (*n* reporting at baseline = 10 [37%], *n* reporting at end of treatment = 3 [11.1%]; paired RD = −1.00, 95% CI: −1.00–−0.33).

**Discussion:**

This program‐led and focused intervention produced meaningful outcomes and circumvented hospitalization for youth at high risk in a short time frame. This approach offers promise for scalable, early ED care that leverages programmatic expertise, consistent with evolving models of mental health service delivery.

**Summary**

- The number of youths hospitalized for eating disorder (ED) treatment has increased dramatically since the onset of the COVID‐19 pandemic, highlighting the need to reduce such hospitalizations via early intervention.
- We conducted a quality improvement initiative aimed at reducing ED‐related hospitalizations in youth at risk for medical instability via frequent visits with adolescent medicine and nutrition providers over an 8‐week period, coupled with focused psychoeducation.
- Our results suggest that outpatient medical management of youth at risk for hospitalization is possible and effective to promote weight gain, reduce disordered weight control behaviors, and avoid hospitalization.

## 1 Introduction

Evidence suggests that the prevalence of eating disorders (EDs) has increased substantially since the onset of the COVID‐19 pandemic. A recent systematic review estimated a 48% increase in ED‐related hospital admissions after the pandemic onset, from 591 observed hospitalizations pre‐pandemic to 876 post‐pandemic onset across 10 studies included (Devoe et al. 2023). Though not uncommon in the course of illness, ED‐related medical hospitalizations pose multiple challenges and burdens to families and health systems. ED‐related hospitalizations focus on acute medical stabilization and management of refeeding complications (Peebles et al. 2017), contrasting with longer‐term ED treatment, which focuses on psychiatric and behavioral healthcare alongside restoration of nutritional status. ED‐related hospitalizations are costly, with an average of $20,817 USD per hospitalization in the United States (Deloitte Access Economics 2020), and have been associated with posttraumatic stress symptoms among caregivers (Timko et al. 2023). Given limitations in hospital capacity (Feldman et al. 2023; Pehlivan et al. 2022), there is an urgent need to reduce the number of youths requiring inpatient medical stabilization, thereby reserving scarce hospital resources for those with the most severe clinical presentations.

Since ED‐related hospitalization for medical stabilization is often lifesaving and cannot be deferred, it is critical to detect youth at risk of hospitalization *before* they exhibit signs of nutrition‐related medical instability, such as bradycardia or electrolyte instability. It has been estimated that up to 10% of ED‐related hospital admissions and 25% of ED‐related emergency department visits could be prevented with prompt outpatient management (de Oliveira et al. 2023). Early intervention has generally been associated with improved outcomes for youth with EDs, such as greater rates of remission at 20‐year follow‐up (Austin et al. 2021). Since evidence indicates that early improvements are predictive of positive long‐term outcomes for youth with EDs (Madden et al. 2015), intervening promptly may reduce the number of hospitalizations needed.

Program‐led and focused models, terms initially coined by Davey, Allen, et al. (2023), have become increasingly popular in the ED early intervention space as they offer a solution that reduces the gap between treatment demand and capacity. “Program‐led” refers to models where the value of the intervention lies in the group or practice providing it, rather than an individual with expertise; “focused” refers to an intervention that requires less capacity than alternatives (i.e., 50% or less therapist time than standard treatment). These models offer a quickly scalable and flexible approach and can play an important role in affording a greater number of people access to ED care (Davey, Bennett, et al. 2023).

To manage the demand‐capacity gap for youth with EDs, it is crucial to design and implement outpatient interventions that successfully reduce the need for medical stabilization, minimizing the number of youths in need of hospitalization. The Rapid Intervention to Support Eating issues (RISE) program was a program‐led and focused intervention designed to treat malnourished, medically stable adolescents nearing hospital admission criteria in an outpatient setting with the goal of nutritional rehabilitation whilst circumventing hospitalization. This work was conducted as a quality improvement initiative, emphasizing systematic efforts to improve clinical processes and patient outcomes within a real‐world practice context. RISE's two overarching goals were to (1) prevent the worsening of illness due to prolonged treatment program wait times and (2) help manage inpatient census. Herein, we describe the RISE model and implementation, as well as preliminary outcomes of the initiative. We include a discussion on barriers and facilitators to the program's implementation, lessons learned, and future directions.

## 2 Methods

### 2.1 Setting

The RISE program was conducted within a large, hospital‐based pediatric care network situated on the east coast of the United States. The care network includes over 30 pediatric primary care sites and a multitude of specialty care sites, three of which offer adolescent medicine care. Specialty care sites also include dietitians, social workers, and in some cases, psychologists. The RISE team included clinicians with ED expertise in adolescent medicine, clinical nutrition, behavioral health, social work, and nursing. The RISE program took place within one of the specialty care sites, which is situated in a suburban district. The catchment area for this site includes predominantly suburban or rural communities. The program was developed collaboratively with all key stakeholders as a quality improvement initiative.

### 2.2 Enrollment

Potential patients were identified in one of three ways: (1) via emergency department providers within the hospital network, (2) via primary care providers within the care network, and (3) via other specialty providers within the network. Patients were eligible for RISE if they met one of the following criteria: heart rate between 50–60 beats per minute or 7.5%–10% weight loss over the last 1–2 months. Inclusion criteria were decided based on institutional admission criteria, which recommend admission with a heart rate below 50 beats per minute or with greater than 10% weight loss in 1–2 months. Thus, these criteria were meant to capture those who were nearing admission criteria but did not presently require admission. Patients were ineligible if they exhibited abnormal electrolyte levels, an abnormal echocardiogram, acutely refused food, or were actively suicidal. A nurse navigator reviewed the records of prospective patients for eligibility and if eligible, contacted the caregiver(s) of the eligible patient to describe the program and timeline. Eligible families who agreed to participate had their initial visit within 7–10 days of their initial presentation.

### 2.3 Patient Flow and Program Structure

**FIGURE 1:**
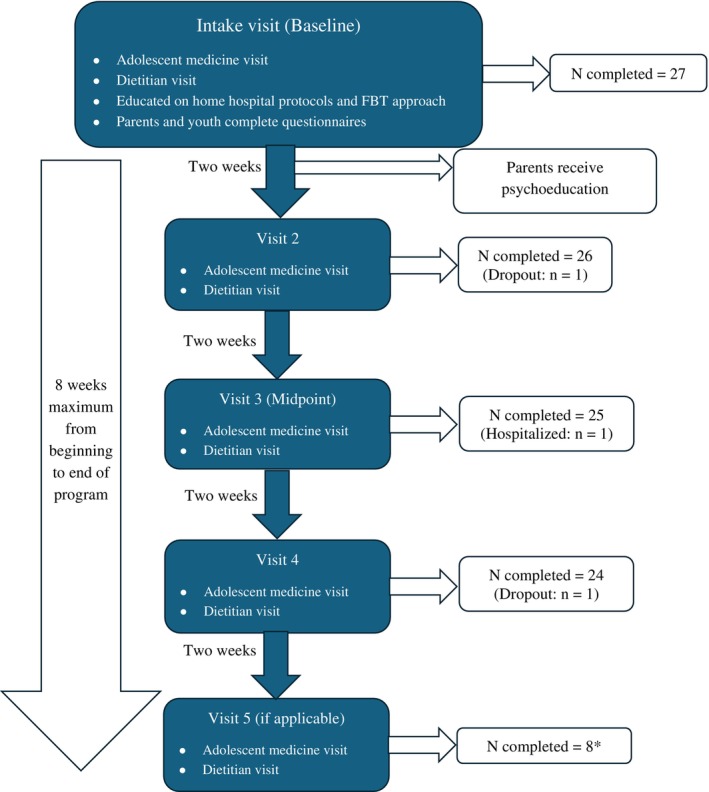
RISE program overview and participant flow. *The RISE program was originally incepted to encompass four visits over 8 weeks. However, when available, some patients were able to attend five bi‐weekly visits within the 8‐week span.

Patients enrolled in the RISE program completed either 4 or 5 visits over an 8‐week period on a bi‐weekly basis between November 2022 and August 2023. Program flow and participation at each timepoint are described in Figure 1. At each visit, patients had appointments with both adolescent medicine and a dietitian. The focus of these visits was informed by Family‐Based Treatment (FBT; Lock and le Grange 2005), with education and care planning centered around phases 1 and 2 of FBT (i.e., empowering parents to prioritize nutritional rehabilitation with the goal of weight restoration). Families were also instructed to implement a “home hospital” protocol, which includes several restrictions, nutritional guidelines, and behavioral guidelines for parents to follow with their adolescent. The home hospital guidelines have been described previously by Peebles et al. (2017), and are listed in Supporting Information.

The adolescent medicine physician focused on providing education about complications associated with malnutrition, monitored for medical stability, supported the family in renourishment, assessed vital signs, and evaluated for any comorbid medical conditions or complications. The dietitian set individualized goal weights and provided recommendations on the rate of weight gain and caloric needs to achieve recommended goals, as well as supported the family with meal planning, calorie boosting, and food exposures. The entire team provided guidance and education on malnutrition, EDs, and supported the family with distress tolerance during the renourishment process. After the completion of the 8‐week RISE session, families were offered “usual care” (i.e., follow‐up with adolescent medicine service as recommended by the provider).

### 2.4 Behavioral Health and Psychoeducation

As part of the quality improvement focus of RISE, the program piloted multiple methods of integrating behavioral health. Importantly, outpatient behavioral health did not have a standing presence in the specialty care setting where RISE took place. All families who participated received a “Welcome Packet” at the time of enrollment, which detailed the program, what to expect during the intake appointment, home hospital protocols, coping strategies for parents and adolescents, behavior management techniques, information about malnutrition in youth, and additional resources such as books and websites. Additionally, we piloted two methods of supporting families. The first was a single‐session evaluation with a psychologist (in person or via telehealth) that focused on evaluation, diagnosis, and preliminary psychoeducation. Halfway through the RISE pilot, we piloted a single‐session group psychoeducation intervention for caregivers of youth enrolled in RISE. The group included information about malnutrition, ED cognitions and behaviors, ED treatment, renourishment, and recovery. Both behavioral interventions were delivered within the first 2 weeks of the RISE program.

### 2.5 Baseline Data Collection and Longitudinal Outcomes

Baseline demographic information included patients' age, sex assigned at birth, gender, race, ethnicity, insurance status (public or private), and referral source. Diagnoses were made in accordance with DSM‐5 criteria at the completion of the initial RISE appointment, which followed a standard format. Families also completed a set of questionnaires at baseline only. Youth completed the 12‐item Eating Disorder Examination—Questionnaire Short (EDE‐QS; Gideon et al. 2016), which is an abbreviated version of the 36‐item Eating Disorder Examination Questionnaire. Importantly, the EDE‐QS asks respondents to reflect on the prior 7 days, as opposed to the 28‐day perspective in the EDE‐Q. Scores on the EDE‐QS can range from 0–36, with 0 indicating no ED pathology and 36 indicating marked ED pathology. The threshold for clinical significance is a score of 15 (Gideon et al. 2016). Caregivers completed the Parent Versus Anorexia (PVA) questionnaire and the Eating and Activity Questionnaire for Parents (EAQP). The PVA is a 7‐item questionnaire meant to assess parental self‐efficacy in caring for youth with EDs (Gorrell et al. 2023). PVA scores range from 5, the lowest parental self‐efficacy, to 35, the highest parental self‐efficacy. The EAQP, a shortened version of the Anorectic Behavior Observation Scale, is comprised of 10 items and assesses ED‐like behaviors observed by parents of youth with EDs or suspected EDs (Thiels and Schmitz 2009). Items are scored 0 (no), 1 (maybe), and 2 (yes); total scores can range from 0 (no ED behaviors) to 20 (marked ED behaviors). It is validated as a population‐level screening tool for suspected EDs when completed by parents.

We piloted multiple different modalities to gather baseline questionnaire data including via email and in‐person (via iPad) in order to determine the most effective method. It was determined that email was more successful than in‐person administration, and thus, most families received questionnaires via email. Still, some families did not complete baseline questionnaires despite reminders. Longitudinal data were collected at three timepoints: Baseline, week 4 (program midpoint) and week 8 (end of treatment). Baseline outcome data included: Caloric intake (as reported by parent); recommended caloric intake; current weight, height, and body mass index (BMI); highest historical (premorbid) BMI‐for‐age; median BMI‐for‐age at baseline; orthostasis (defined as a difference of greater than 20 BPM by heart rate and/or 10 mmHG by blood pressure from sitting or standing); patient report of disordered weight control behaviors (binging, purging, laxative abuse, diet pill use, compulsive exercise, as reported by patient and parent during clinical interview with dietitian); and clinician‐recommended goal weight range. Compulsive exercise was considered exercise that was either secretive or against medical recommendation. We also collected data on non‐compulsive exercise when appropriate, which included activities such as walking, physical education class, or organized sport participation, with provider clearance. Goal weights were determined based on ideographic expected body weight, using developmental weight suppression calculation (Singh et al. 2021).

Week 4 (midpoint) data collection included weight, BMI, and median BMI‐for‐age.

Week 8 (end of treatment) data collection included all baseline data as well as the percentage of goal weight achieved and whether hospitalization or referral to a higher level of care occurred during the RISE program. From a programmatic perspective, we also assessed rates of completion of the program and the rate of enrollment in the program.

### 2.6 Protection of Human Subjects

Given that this program was conducted as a quality improvement initiative, the conduct of the program was not considered human subjects research. However, analysis and publication of the quality improvement data were reviewed by the health system Institutional Review Board, and the work was deemed to be exempt.

### 2.7 Analysis and Data Availability

Descriptive statistics were used to report baseline demographic and questionnaire data, as well as group‐level data at each time point. Changes in the prevalence of disordered weight control behaviors from baseline to week 8 were evaluated with a paired t‐test and effect sizes were estimated with paired‐sample Cohen's *d* (*dz*). Changes in the prevalence of orthostasis and reported caloric intake were evaluated with McNemar's test and paired risk differences. Linear mixed‐effects models were used to estimate the longitudinal change in body weight, percentage of median BMI‐for‐age (%mBMI), and percentage of low‐ and high‐end of goal weight range attained. All analyses were conducted in R Studio version 2025.5.1. Original, de‐identified data from this quality improvement initiative are available upon reasonable request.

## 3 Results

The 27 patients who participated in the RISE program are described in Table 1. An additional 12 patients were referred for evaluation but did not participate. Of these patients, 8 (66.6%) declined to participate, 3 (25%) were unreachable, and 1 (8.3%) was determined to be ineligible for the program. Throughout the program's implementation, two youth dropped out (7.4%), and one was hospitalized (3.7%). Patients were predominantly female (*n* = 23, 85.2%), White (*n* = 20, 74.1%), and non‐Hispanic/Latinx (*n* = 20, 74.1%). Most patients (*n* = 14, 51.8%) carried public insurance. Patients varied in diagnosis; the most common diagnosis was anorexia nervosa‐restricting type (AN‐R; *n* = 8, 29.6%). The mean age was 14.5 years (SD = 2.4 years).

**TABLE 1:** Patient demographics and baseline characteristics.

Characteristic	*N* (%)
Overall sample	27
Dropout	2 (7.4)
Hospitalized during intervention	1 (3.7)
Sex assigned at birth	
Female	23 (85.2)
Male	4 (14.8)
Race	
Asian/Pacific Islander	1 (3.7)
Black	1 (3.7)
White	20 (74.1)
Other or mixed race	5 (18.5)
Ethnicity	
Hispanic/Latinx	7 (25.9)
Not Hispanic/Latinx	20 (74.1)
Insurance status	
Public insurance	14 (51.8)
Private insurance	13 (48.1)
Diagnosis^a^	
AN‐R	8 (29.6)
AN‐BP	3 (11.1)
AAN	3 (11.1)
BN^a^	3 (11.1)
UFED	7 (25.9)
ARFID	3 (11.1)
Referral source	
Emergency department	8 (28.5)
Primary care	16 (59.2)
Specialty care	2 (7.4)
Direct (caregiver contacted adolescent medicine)	1 (3.7)

Characteristic	M (SD)
Anthropometrics	
Premorbid BMI percentile^b^	64.5 (27.6)
BMI percentile at baseline	28.4 (26.2)
Weight suppression at baseline^c^	−36.6 (20.7)

^a^ Diagnoses reported based on DSM‐5 criteria. Thus, patients appearing to meet criteria for AAN but who also exhibited binging and purging were diagnosed with BN for the purposes of these analyses.

^b^ Historical weight information was unavailable for one patient.

^c^ Weight suppression calculated as change in BMI percentile from highest historical BMI percentile to baseline BMI percentile.

Diagnoses are reported based on DSM‐5 criteria. Thus, three patients appeared to meet criteria for atypical anorexia nervosa (AAN) but also exhibited binging and purging. Though clinically, these patients were managed as AAN patients with binge/purge tendencies, they more closely met DSM‐5 criteria for bulimia nervosa (BN) and thus were diagnosed with BN for the purposes of these analyses. Some degree of weight suppression was consistently present in RISE patients, though the degree of weight suppression varied. The average BMI percentile at baseline was 28.4 (SD = 26.2), and the average patient had a reduction of 36.6 (SD = 20.7) in their BMI‐for‐age percentile from their highest premorbid percentile to baseline. The most common referral source was via primary care providers, who accounted for 59.2% (*n* = 16) referrals.

Sixteen adolescents (59.3%) completed the EDE‐QS. The mean score was 12.6 (SD = 10.1). Nineteen parents (70.3%) completed the PVA with a mean score of 19.1 (SD = 3.33). Finally, 18 parents completed the EAQP with a mean aggregate score of 0.83 (SD = 0.41).

Baseline to end‐of‐program outcomes are described in Table 2. At baseline, patients consumed an average intake of 1252.5 kcal, or 43.3% of their recommended kcal intake. At week 8, patients consumed an average intake of 2274 kcal, or an average of 76% of their recommended intake. A paired *t*‐test showed a statistically significant difference between baseline caloric intake and caloric intake at week 8 with a large effect (M_diff_ = 0.29, *dz* = 0.98, 95% CI 0.45–1.50). The proportion of youth endorsing at least one disordered weight control behavior significantly decreased from baseline (*n* = 10, 37%) to post (*n* = 3, 11.1%; paired risk difference [RD] = −1.00, 95% CI −1.00–−0.33). No patients newly endorsed disordered weight control behaviors at end‐of‐program, and nine no longer endorsed. Additionally, 13 youth reported participating in some form of non‐compulsive exercise (i.e., walking, modified sports) at week 8. The proportion of youth exhibiting orthostasis at baseline (*n* = 10, 37%) to post (*n* = 9, 33.3%) decreased marginally, but the difference was not statistically significant (paired RD = 0.00, 95% CI −0.68–0.68).

**TABLE 2:** Baseline‐to‐end of treatment outcomes.

	Baseline M (SD)	Week 8 M (SD)	*dz*	95% CI
Reported % of minimum calorie recommendation consumed^a^	43.3 (18.3)	76.0 (28.0)	0.98	[0.45–1.50]

	Baseline *N* (%)	Week 8 *N* (%)	Paired RD	95% CI
Reported at least 1 disordered weight control behavior	10 (37.0)	3 (11.1)	−1.00	[−1.00–0.33]
Positive orthostatic vital signs (by heart rate or blood pressure)	10 (37.0)	9 (33.3)	0.00	[−0.68–0.68]

^a^ Based on 24‐h dietary recall.

Linear mixed‐effects models were used to describe the change in mean weight, percent of the median BMI‐for‐age, percent of low end of goal weight range attained, and percent of high end of goal weight attained. Models are described in Table 3. We noted significant improvements over all time points across all four models, with greater improvement between baseline and midpoint than between midpoint and end of treatment. Mean weight increased significantly from baseline to midpoint (+2.21 kg, 95% CI: 1.3–3.1) and from baseline to end of treatment (+3.64 kg, 95% CI: 2.6–4.7). This weight increase translated to a mirrored increase in %mBMI‐for‐age, with an average increase of 4.01% from baseline to midpoint (95% CI: 1.6%–6.5%), and 7.59% from baseline to end of treatment (95% CI: 5.1–10.1). Percent attained of low end of goal weight range increased by 4.38% from baseline to midpoint (95% CI: 2.4–6.3) and by 7.1% from baseline to end of treatment (95% CI: 5.1–9.1), and similarly, percent attained of high end of goal weight range increased by 4.11% from baseline to midpoint (95% CI: 2.3–5.8) and by 6.49% from baseline to end of treatment (95% CI: 4.7–8.3).

**TABLE 3:** Linear mixed‐effects models for weight‐related variables.

Outcome	Baseline (M)	Δ Baseline—Week 4	95% CI	Δ Week 4—Week 8	95% CI	Δ Baseline—Week 8	95% CI
Weight (kg)	45.7	+2.21	1.3–3.1	+1.43	0.5–2.3	+3.64	2.6–4.7
%mBMI	91.1	+4.01	1.6–6.5	+3.58	1.1–6.1	+7.59	5.1–10.1
% Low Goal Weight	84.33	+4.38	2.4–6.3	+2.7	0.73–4.7	+7.10	5.1–9.1
% High Goal Weight	76.85	+4.11	2.3–5.8	+2.39	0.58–4.2	+6.49	4.7–8.3

*Note*: Estimates represent fixed effects from linear mixed‐effects models with a random intercept for participant. Values reflect mean weight at baseline and within‐participant changes at Weeks 4 and 8.

## 4 Discussion

In this quality improvement initiative, we examined the impact of an 8‐week program‐led and focused intervention for youth with eating disorders and malnutrition on hospitalization rates, weight gain, caloric intake, and disordered weight control behaviors. Throughout the duration of the program, one patient was hospitalized (3.7%). We found a significant improvement across all reported weight metrics (weight, %mBMI‐for‐age, percent attained of low end and high end of goal range), with the greatest improvement in the initial weeks (between baseline and week 4). Patients reported consuming significantly more of their calorie goal at week 8 than at baseline, and we noted substantial reductions in the report of disordered weight control behaviors between baseline and week 8. We also noted a very small, likely negligible (*n* = 1) decrease in the prevalence of positive orthostatic vital signs between the beginning and end of treatment.

Most patients who began the initiative completed the entire 8‐week program, with 7.4% (*n* = 2) dropping out and 3.7% (*n* = 1) becoming hospitalized during the program. Although not a formal measure of acceptability, the low rate of attrition that we observed indicates that patients and families did find the program worthwhile. The sample was predominantly female, White, and non‐Hispanic. Although the demographic composition was relatively homogenous, this pattern is consistent with prior retrospective and naturalistic evaluations of outpatient care for EDs (Forman et al. 2011, 2014). Patients varied by diagnosis, indicating that RISE may be effective transdiagnostically. Due to the small sample size, we were unable to determine whether outcomes were different between diagnosis groups.

The mean EDE‐QS score of 12.6 was below the documented clinical threshold of 15 (Gideon et al. 2016). However, it is important to note that our sample varied widely by EDE‐QS score (SD = 10.1), and that the EDE‐QS was not originally validated to detect or evaluate youth with UFED or ARFID, which comprised 40% of our sample. Parents displayed a mean score of 19.1 on the PVA questionnaire, which is consistent with the findings of Gorrell et al. (2023), who examined parents at the beginning of ED‐related hospitalization. Of the 10 items on the EAPQ, parents frequently endorsed three: “My child seldom mentions being hungry,” “My child studies or works diligently,” and “My child claims to be normal, healthy, or even better than ever.” As a screening tool, an average EAPQ score of 1.0 indicates risk for an eating disorder, as parents typically do not endorse items for youth who do not have/are at risk for an ED. While the average score for the group was less than 1.0, 44.4% had an average score greater than 1 and all caregivers endorsed at least one item.

At baseline, the sample averaged 91.1% of the median BMI for age. However, it should be noted that %mBMI varied significantly among the sample (SD = 12.1), and that on average, patients presented to RISE at 36.6 percentiles below their highest premorbid weight, indicating consistent developmental weight suppression. Further, the average participant weighed only 76.8% of the high end of their goal range.

Patients showed statistically significant weight gain throughout the program, with more weight gain concentrated in the first half of the program (from baseline to week 4). This is encouraging, given that evidence suggests that early weight gain (and ED symptom improvement) is associated with improved treatment outcome (Le Grange et al. 2014; Vall and Wade 2015). Weight gain slowed but continued over time, with smaller but still statistically significant improvements between weeks 4 and 8 as well. A continued increase in weight gain in the latter half of the program suggests that the duration of 8 weeks may be appropriate, though more research is needed to determine the most appropriate “dose” of the RISE program.

As expected, patients consumed significantly more calories between baseline and week 8 of the program. Given that the RISE program was FBT‐informed, parents were asked to take control of adolescents' eating and intake, beginning at visit 1 (Rienecke 2017). Our findings suggest that parents were generally able to increase the number of calories their child consumed, and that education delivered via physician, dietitian, and psychoeducation can aid families in developing appropriate meal plans to promote weight gain.

The number of youths who reported disordered weight control behaviors significantly decreased throughout the duration of the program, from 10 at baseline to 3 at week 8 (paired RD = −1.00; 95% CI: −1.00–−0.33). Wons et al. (2023) noted a similar decrease in maladaptive exercise among youth with EDs after a 12‐week cognitive behavioral therapy intervention, suggesting that focused or time‐limited interventions may be effective at minimizing such behaviors. Though families were educated on home hospital protocols which include minimal movement at the beginning of RISE, many had achieved enough weight gain and medical stability to be cleared by the RISE team to return to some form of physical activity.

The number of youth who were orthostatic by heart rate and/or blood pressure decreased by only one patient (from *n* = 10 at baseline to *n* = 9 at week 8). Though the improvement is marginal and not significant, it was not unexpected given the prolonged nature of cardiac recovery post‐malnutrition (Powers et al. 1991).

One finding of interest is that most patients participating in RISE did not receive specialized behavioral health care for malnutrition. While behavioral health assessment and psychoeducation were a part of the RISE program, longitudinal therapy was not. Though parents were given a list of FBT providers, formal referrals for a behavioral health evaluation were not made. Some patients in RISE were already receiving behavioral health treatment in the community (e.g., supportive treatment for anxiety); none of these were receiving eating disorder‐focused care. Future iterations of RISE, perhaps without the behavioral health component, may be beneficial to determine if the same degree of success is feasible without behavioral health involvement. Having an even more limited role of a behavioral health provider may increase translatability to other practice settings, as most practice locations do not have embedded psychologists.

Overall, the RISE program was successful in preventing hospitalization among most at‐risk youth with eating issues. Participation in the program resulted in increased caloric intake, weight gain, and reduction in disordered weight control behaviors. However, these results should be interpreted in the context of the purpose of RISE. Given that RISE was a quality improvement initiative, we did not recruit a control or “usual care” group with which to compare outcomes, and comparison between participants and non‐participants was not possible. Similarly, the quality improvement context does not require strict protocol adherence or high intervention fidelity. As a result, some deviations from the stated protocols and recommendations likely occurred in response to the complexities of delivering care in a real‐world clinical setting. Additionally, as most RISE patients were new to the RISE team providers and newly developing patient‐provider rapport, social desirability bias may have impacted patient reports of disordered weight control behaviors, calorie intake, or questionnaire responses. We attempted to mitigate this by relying on parent reports whenever possible. Finally, the demographic homogeneity of the sample suggests that these findings may not be generalizable to a larger sample.

We also encountered several barriers to the program's implementation that may impact future iterations of RISE or its uptake in other settings. First, the program's embedding within a large health system meant that many families traveled from out of state or from considerable distances to receive care. Thus, scheduling consistent biweekly visits became difficult in some cases. Similarly, as the program was new, a limited number of providers offered RISE visits, and thus, there were a limited number of appointment slots per week. Given that a central intention of the program was to increase care access, future iterations of RISE may include a greater number of providers to further expand access. We also experienced limited response rates to questionnaires and administered them only at baseline to minimize patient burden. For future iterations of RISE, we plan to integrate questionnaires into the electronic health record to allow for more seamless administration and administer them at each visit. Finally, it was challenging to inform all potential referral sources of the RISE program and enrollment criteria (both within the health system and external to it), as the health system encompasses such a large catchment area.

Future work should examine outcomes of the RISE program with larger samples, and in varied contexts (i.e., other health systems and settings such as primary care and community settings). Further, RISE outcomes should be compared with control, waitlist, or usual care outcomes to allow for meaningful understanding and interpretation of improvements above those that are expected. Given that the preliminary effectiveness of RISE has now been established, future studies may focus jointly on effectiveness and implementation to create a scalable and adaptable intervention for potential wide‐scale use.

## 5 Conclusion

Since the onset of the COVID‐19 pandemic, ED hospitalizations have risen sharply, underscoring the urgent need for scalable outpatient interventions that can prevent severe illness and reduce strain on limited hospital resources. In this paper, we evaluate a program‐led and focused quality improvement initiative, RISE, designed to identify and manage youth at risk of ED‐related hospitalization. Patients who participated in RISE exhibited weight gain at all timepoints, increased caloric intake, and decreased prevalence of disordered weight control behaviors. Evaluation of RISE indicates that weight restoration can be achieved in 8 weeks without hospitalization with a program‐led and focused intervention. Further, we have demonstrated that behavioral health support can be provided without an individual approach, rather than psychoeducation may be successful, particularly in settings where behavioral health support is limited. Finally, our findings illustrate that families can successfully be taught and implement the principles of FBT in an outpatient, medically focused setting. Future research should evaluate RISE in larger and more diverse settings, using comparison groups to assess its added benefit, and shift toward hybrid effectiveness‐implementation designs to support scalability.

## Conflicts of Interest

The authors declare no conflicts of interest.

## Supporting information


**Data S1:** Supporting Information.

## Data Availability

The data that support the findings of this study are available from the corresponding author upon reasonable request.
