# Supplementary material for: Rapid Intervention to Support Eating Issues (RISE) Program: Using Quality Improvement to Reduce Medical Hospitalization in Malnourished Youth
Source: Int J Eat Disord. 2025 Nov 18;59(3):501–9. doi: 10.1111/eat.70004 (PMC12979960; doi:10.1111/eat.70004)
Supplement: Supplementary file 1 — Data S1: Supporting Information. [file EAT-59-501-s001.docx]

**Supplementary Material A**

| **RISE Nutrition Rehabilitation Pathway Behavioral Health Home Hospital Plan** | |
| --- | --- |
| **Meal Guidelines** | |
| **Caregivers are responsible for:**   - Grocery shopping - Menu planning - Preparation of all meals and snacks - Supervision of all meals, snacks and rest periods      \| Timing: 30 minutes for meals, 15 minutes for snacks, 15 minutes to supplement  Rest Period: Required physical rest for 1 hour after meals and 30 minutes after snacks.  No bathroom use permitted during this time unless direct supervision is provided.    Follow meal and snack schedule 7 days/week.    **Child/Teen:**   - Complete 100% of meals, snacks, and supplement (if needed) - No negotiation about food choices or amounts \| \| --- \| | |
| **Physical Activity Guidelines** | |
| \| - Only light activity within the home is permitted - No exercise or sports activity is permitted - Remain in common living area during the day - Bedroom is for sleeping only - Bathroom should be used only before eating - Showers only in the AM before eating, no longer than 10 minutes - Completing schoolwork is acceptable if it does not interfere with eating - Return to school requires approval from medical and other treatment provider(s) \| \| --- \| | |
| **Things to Do:** | **Things NOT to Do:** |
| - Follow up with medical provider as recommended - Follow up with behavioral health provider as recommended - Follow up with dietitian as recommended | \| - Avoid diet food, caffeine, chewing gum. - Use of weight scales is not permitted. All scales should be removed or hidden. - Access to websites/television shows/movies/social media focused on food, cooking, weight, or shape is not allowed \| \| --- \| |
| **Behavior Guidelines** | |
| **Meals and Snacks:**   - No negotiating meals or snacks - No conversations about calories, weight or other numbers - No comments about food or appearance - Use encouraging statements (“I love you,” “I know you can do this,” “I believe in you”) - Keep conversations low stress and positive - Encourage mechanical eating when needed - Redirect unwanted behavior with brief matter-of-fact statements (“I would like you to stop cutting your food so small,” “Let’s take bigger bites”) - Provide occasional reminders about timing | If child/teen refuses to eat or engages in eating disorder behaviors (such as exercise, vomiting, hiding food), consider the following possible consequences:   - Consider removing physical activity. If your child is not eating, they need to conserve energy. - Consider removing sources of stress such as: - Cell phone - Computer - Social visits - School - Television - Social Media - Other: - Increase supervision by: - Keeping the bathroom door open - Sleeping in the same room - No alone time permitted |
| **Reminders about Effective Behavior Change** |  |
| **Positive reinforcement strategies to increase wanted behaviors:**   \| - Praise - Motivating rewards (such as: visits with friends, screen time) - “First ________, then _________.” - Make sure the size of the reward fits the size of the good behavior - Natural and logical consequences help your child to make better decisions - Remember to work together with other caregivers \| \| --- \| | |
| **Healthy Coping Strategies that I Will Role Model** | |
| **Possible suggestions:**   - Take a break when I am feeling angry or upset - Name my feelings out loud (“I feel ___”) - Communicate my needs (“I need _____”) - Self-soothe using my senses - Be willing to ask for help when I need it - Take care of my own feelings and needs - Apologize when I do something wrong - Positive affirmations or mantras (“I can do this”) - Use “I” statements instead of “you” statements - Schedule “me” time - Be consistent and reliable - Practice gratitude - Get fresh air and remember to **BREATHE!** | |
